# Differences in the Aerobic Capacity of Flight Muscles between Butterfly Populations and Species with Dissimilar Flight Abilities

**DOI:** 10.1371/journal.pone.0078069

**Published:** 2014-01-08

**Authors:** Virve Rauhamäki, Joy Wolfram, Eija Jokitalo, Ilkka Hanski, Elizabeth P. Dahlhoff

**Affiliations:** 1 Helsinki Bioenergetics Group, Structural Biology and Biophysics Program, Institute of Biotechnology, University of Helsinki, Helsinki, Finland; 2 Metapopulation Research Group, Department of Biosciences, University of Helsinki, Helsinki, Finland; 3 Electron Microscopy Unit, Institute of Biotechnology, University of Helsinki, Helsinki, Finland; 4 Department of Biology, Santa Clara University, Santa Clara, California, United States of America; USDA-Agricultural Research Service, United States of America

## Abstract

Habitat loss and climate change are rapidly converting natural habitats and thereby increasing the significance of dispersal capacity for vulnerable species. Flight is necessary for dispersal in many insects, and differences in dispersal capacity may reflect dissimilarities in flight muscle aerobic capacity. In a large metapopulation of the Glanville fritillary butterfly in the Åland Islands in Finland, adults disperse frequently between small local populations. Individuals found in newly established populations have higher flight metabolic rates and field-measured dispersal distances than butterflies in old populations. To assess possible differences in flight muscle aerobic capacity among Glanville fritillary populations, enzyme activities and tissue concentrations of the mitochondrial protein Cytochrome-*c* Oxidase (CytOx) were measured and compared with four other species of Nymphalid butterflies. Flight muscle structure and mitochondrial density were also examined in the Glanville fritillary and a long-distance migrant, the red admiral. Glanville fritillaries from new populations had significantly higher aerobic capacities than individuals from old populations. Comparing the different species, strong-flying butterfly species had higher flight muscle CytOx content and enzymatic activity than short-distance fliers, and mitochondria were larger and more numerous in the flight muscle of the red admiral than the Glanville fritillary. These results suggest that superior dispersal capacity of butterflies in new populations of the Glanville fritillary is due in part to greater aerobic capacity, though this species has a low aerobic capacity in general when compared with known strong fliers. Low aerobic capacity may limit dispersal ability of the Glanville fritillary.

## Introduction

Flight is critically important for the fitness of most insects due to its role in reproduction [1]–[3] and dispersal [4]–[7]. While the relationship between flight ability and dispersal is evident, the corresponding physiological mechanisms are not well understood for most insects, the exception being wing dimorphic species [8], [9]. What is known is that flapping flight is one of the most energetically expensive forms of locomotion [10] and thereby likely to involve strong selective constraints. Insect flight muscle is metabolically very active: flight metabolic rate may exceed resting metabolic rate by two orders of magnitude and accounts for most of the whole-body oxygen consumption in flying insects [11]–[13]. In hummingbirds, bats, and several species of insects, flight performance is highly correlated with indicators of the aerobic capacity of flight muscle, such as mitochondrial respiration, enzyme activities, and mitochondrial size and concentration [13]–[18]. These biochemical indicators are invaluable for assessing aerobic capacity of species that cannot be easily studied in a laboratory setting, making them useful metrics for studying dispersal ability in nature.

The Glanville fritillary butterfly (*Melitaea cinxia* L.) has become a model species for studies of dispersal in fragmented landscapes [19],[20]. Comprehensive investigations of a large metapopulation in the Åland Islands in Finland over the past two decades have revealed significant differences between individuals and local populations in dispersal ability [21], [22] and flight metabolic rate [4], [5], which influence the establishment of new local populations [23]. Individuals with the highest flight metabolic rates in the laboratory have the longest flight distances per unit time in the field [5], and these traits are highly heritable [24]. In nature, butterflies in newly-established local populations have higher mobility and flight metabolic rates than butterflies in old local populations, apparently because new populations are established by more dispersive females than the average female in the metapopulation [4], [21], [25]–[28].

Previous studies have also shown that a single nucleotide polymorphism (SNP) in the coding region of the gene phosphoglucose isomerase (*Pgi*) is significantly associated with variation in flight metabolic rate and dispersal rate in the field [4], [5]. Specifically, heterozygotes in SNP *Pgi_111* have better flight performance over a broader range of environmental temperatures than homozygotes [5], [26], [27]. PGI is a glycolytic enzyme critical for carbohydrate metabolism during flight, and the gene *Pgi* encoding for this enzyme has been shown to be under natural selection in a number of insect species [29]–[36], including the Glanville fritillary [20], [37]–[39]. An association between *Pgi* genotype, metabolic rate and flight performance implies a significant role for glycolysis in the dispersal of the Glanville fritillary.

Aerobic metabolism is crucial for fuelling both short- and long-distance flights necessary for successful dispersal [40]–[43]. The Glanville fritillary is an ideal species to study this problem, as a role for adaptive variation in enzymes of glycolysis for dispersal has already been established. Activity of glycolytic enzymes may be directly linked to activity of aerobic metabolic pathways, because carbohydrates fuel short-term flight muscle activity in many small insects [41], [43]–[46], and do so exclusively in the Glanville fritillary (RQ = 1 for flights of 10 min; [47]. Functional differences among the PGI allozymes may result in differences in rates of carbon flow through glycolysis, which may limit the rate of ATP production by flight muscle mitochondria [34], [48], [49]. Recent studies suggest that there are functional links between *Pgi* and other metabolic genes, including mitochondrial genes coding for proteins important for regulating oxygen sensing, and genes coding for Cytochrome *c* oxidase, the enzyme that catalyzes the reduction of oxygen to water in cellular respiration [47], [50], [51]. Alternatively, differences in aerobic capacity could be directly related to differences in mitochondrial concentration and aerobic enzyme activity, independent of glycoysis [52]. Either way, examination of variation in aerobic capacity among dispersal phenotypes is critical for a better understanding of how aerobic capacity may contribute to dispersal ability of species in fragmented landscapes.

To place intra-specific variation in the aerobic capacity of the Glanville fritillary into a broader ecological context, we measured aerobic capacity of four other species of Nymphalid butterflies. We were not able to measure flight metabolism or dispersal distances in these specimens, and hence selected species with clearly contrasting flight behaviours and dispersal phenotypes based on literature and personal observations (summarized in Table 1). Three species are powerful fliers with historically broad biogeographic ranges and effective population sizes: *Aglais urticae* (Small Tortoiseshell), *Argynnis adippe* (High Brown fritillary), and *Vanessa atalanta* (Red Admiral). In contrast, the fourth additional species, *Aphantopus hyperantus* (Ringlet) and the Glanville fritillary fly only short distances during individual flight bouts (Table 1). To compare aerobic capacity of flight muscle among populations and between species, we used polarographic determination of oxygen consumption rate of the mitochondrial enzyme Cytochrome *c* Oxidase (CytOx) and optical spectroscopy to measure CytOx content in flight muscle of all five species. We also used electron microscopy to compare the morphology of flight muscles of one species that flies short distances and one species that flies long distances.

**Table 1:** Differences in body size and ecological characteristics of the Nymphalid butterflies compared in this study.

*Species* (Common name)	Thorax fresh mass (mg)	Thorax protein content (mg/g)	Population dynamics	Flight activity and behaviour during mating season
*Aphanatopus hyperantus* (Ringlet)	15±3	62±3	Isolated populations, limited dispersal; individuals move less than 100 m per day^1^	Short flight bouts in males and females; males patrol for perching females.^2^
*Melitaea cinxia* (Glanville fritillary)	33±1	52±1	Metapopulation;1–3 km life-time dispersal range, depending on population size and fragmentation^3^	Males patrol for eclosing females in short flight bouts; females fly farther than males.^4^
*Aglais urticae* (Small Tortoiseshell)	59±3	56±3	Long-distance migrant; genetic connectivity between Southern and Northern Europe^5^	Powerful flight in males and females; males territorial and highly aggressive.^6^
*Argynnis adippe* (High Brown fritillary)	81±3	48±3	Formerly common with large geographic range; fragmented populations due to habitat loss^7^	Powerful flight in males and females; travel >1 km during mating season^8^
*Vanessa atalanta* (Red Admiral)	110±2	67±3	Long-distance migrant; genetic connectivity along north-south migration corridors^9^	Powerful flight in males and females; males territorial and highly aggressive^10^

**References.**

^1^ . [59]–[61];

^2^ . [62];

^3^ . [20], [63]–[65];

^4^ . [20], [22], [23];

^5^ . [66], [67];

^6^ . [68];

^7^ . [69], [70];

^8^ . [68], [70];

^9^ . [71]–[73];

^10^ . [74].

## Materials and Methods

### Sample collection

Glanville fritillary caterpillars were collected in the spring of 2008 from 43 different local populations in the Åland Islands in Finland [23], [53]. Dispersing females had established 26 of the 43 populations in the previous year, and hence in these cases we sampled the offspring of the original colonizers, whereas 17 populations were at least 5 years old; the age of these populations is known due to long-term monitoring of the entire metapopulation [23]. Caterpillars were reared into butterflies in common garden conditions that mimicked summer field conditions (16 h 28°C d, 8 h 20°C night) and fed *ad libitum* on the host plant *Plantago lanceolata*. One or two butterflies per local population were selected for the analyses (*n* = 73 total). One-day old butterflies were used for sample preparation (below). To test for possible effect of prior flight activity on the measurements we analysed 19 additional butterflies at the age of 5 days in 2009. These butterflies were maintained in cylindrical cages (40×50 cm) at 20/28°C (night/day, 16∶8 hrs). Half of the butterflies were kept under bright light to encourage active flight, while the rest were kept in darkness and the butterflies were hence completely inactive following their eclosion. For comparisons among species with different flight behaviours, newly-eclosed adults were collected in Southern Finland in the summer of 2008, dissected immediately and flash-frozen in liquid nitrogen. Newly-eclosed Red Admirals (*V. atalanta*) used in the electron microscopy study were collected in Catalonia in fall 2008 and sent alive to the laboratory for sample processing. No permits were required for the described study, which complied with all relevant regulations.

### Sample preparation

All chemicals were obtained from Sigma-Aldrich (St. Louis, Missouri, USA) except *n*-dodecyl *β*-D-maltoside (DDM), obtained from Anatrace (Maumee, Ohio, USA). Butterflies were cooled in a refrigerator before dissection. Whole thoraces were dissected, weighed, flash-frozen in liquid nitrogen and stored at −80°C until biochemical analyses. To measure enzyme content and activity, the entire frozen thorax was sliced with a scalpel and immersed in 700–1000 µl of ice-cold homogenization buffer (50 mM potassium phosphate, pH 6.5 @ 22°C, 0.05% DDM); thorax tissue (more than 80% of which is flight muscle) was homogenized at 4°C in an ice-cold ground glass homogenizer. The insoluble pellet, composed primarily of chitin, was discarded after centrifugation for 10 min at 14,000 rpm, 4°C (5417R micro-centrifuge, Eppindorf Nordic, Copenhagen, Denmark). The volume of the resultant supernatant was measured, and homogenates were kept on ice until enzyme activities and concentrations were determined. CytOx activity of the pellet was assayed (details below) to ensure that mitochondrial membranes were solubilised. No residual enzyme activity was measured.

### Cytochrome-c Oxidase activity and concentration

Enzymatic activity of Cytochrome-*c* Oxidase (E.C. 1.9.3.1; CytOx) was determined by measuring oxygen consumption at 25°C using a Clark-type polarographic oxygen electrode (Oroboros Oxygraph, Paar Physica, Austria). Assays were run in duplicate in a buffer containing 50 mM potassium phosphate (pH 6.5 @ 22°C), 0.05% DDM supplemented with 3 mM potassium ascorbate, 0.6 mM N,N,N′,N′-tetramethyl-*p*-phenylenediamine, and 30 µM horse-heart Cytochrome-*c*. Preliminary assays were conducted to insure Cytochrome-*c* concentration was well above saturation. The rate of auto-oxidation was recorded each time and subtracted from the final activity. A sample of 1–5 µl (final concentration of CytOx, 1–2 nM) was used to initiate the reaction, and the maximum reaction rate was recorded. A small amount of potassium cyanide, an inhibitor of CytOx, was added at the end of an assay to determine the rate of non-specific oxygen consumption, as well as to ensure that CytOx was the only oxygen-consuming enzyme in the sample. Measured activity was found to be fully sensitive to cyanide; that is, no residual activity above the level of auto-oxidation was observed after inhibition of CytOx.

**Figure 1:**
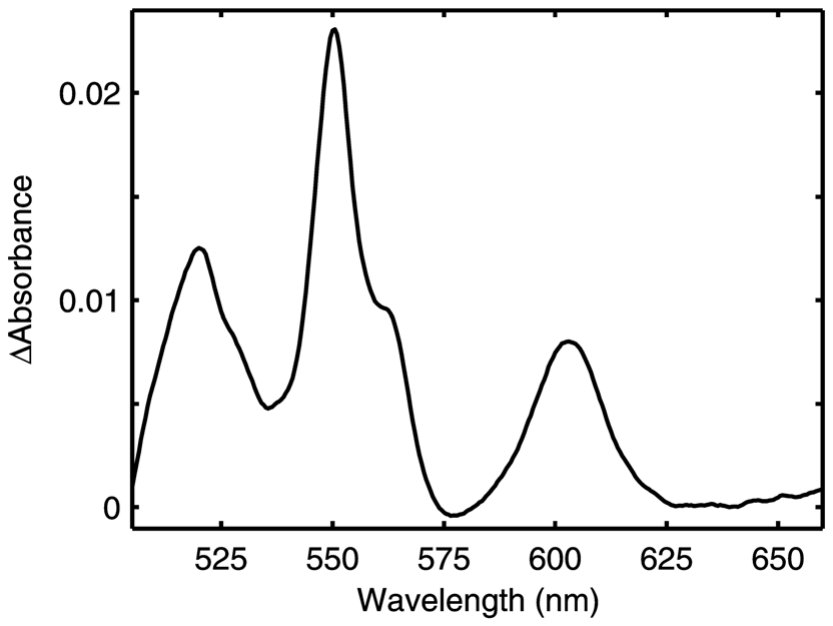
Reduced *minus* oxidized difference spectrum of a butterfly thorax preparation. Optical spectra of the flight muscle homogenate were collected in the ferricyanide oxidized and the dithionite reduced states. The difference spectrum has clear peaks at 550, 560 and 605*c*, *b* and *a*, respectively. This method allows highly accurate determination of tissue concentrations of the respiratory protein CytOx (also known as cytochrome *aa_3_* or Complex IV).

Tissue concentrations of CytOx were determined spectroscopically following published methods [17]. All spectra were run at 25°C using a Shimadzu 3000 UV-VIS spectrophotometer (Shimadzu Corporation, Kyoto, Japan). A representative spectrum is presented in Fig. 1. Samples were oxidised using ferricyanide and reduced by dithionite. CytOx concentrations were calculated from the reduced *minus* oxidised difference spectra values using the Lambert-Beer relationship (c = A/ε, path length at 1 cm). Extinction coefficients (ε) used for the wavelength pairs 550 nm *minus* (535 nm+575 nm)/2 (cytochrome *c*) and 605 nm *minus* (580 nm+630 nm)/2 (cytochrome *aa_3_*) 605 nm *minus* (580 nm+630 nm)/2 were 19 mM^−1^ cm^−1^ and 27 mM^−1^ cm^−1^, respectively. The average ratio of Cytochrome-*c* to CytOx was 4.2. To control for differences in body size, CytOx activity and concentration values were normalized to tissue soluble protein, determined using a modified Lowry assay (BCA Reagent, Pierce Biotechnology, Rockford, IL, USA).

Statistical analyses were conducted using Jmp Pro 10 (SAS Institute Inc. Cary, NC, USA). Five statistically significant (*P*<0.01) outliers were removed from the data set for the Glanville fritillary (*n* = 2 for CytOx activity, *n* = 3 for CytOx content). Data were otherwise normally distributed. Differences between Glanville fritillary populations were compared using one-way ANOVA. Males and females did not differ in mass-specific enzyme activity or content, so sex was not included in the final analysis. For differences between species, variances were not equal due to unequal sample sizes. While this is not typically an issue for low order ANOVAs when *P* values are low [54], we grouped species by known flight behaviour (long-distance or strong fliers versus short-distance fliers) and ran a nested ANOVA, with species nested in flight type, to examine these data.

### Pgi genotyping

A single nucleotide polymorphism (SNP) was genotyped in the coding region of the gene phosphoglucose isomerase (*Pgi_111*) using the procedure described by Orsini et al. [55]. This SNP has been shown to be associated with variation in flight metabolic rate [4], dispersal rate in the field [5], and other key life history traits [23].

### Flight muscle morphology

Three individuals each of the Red Admiral and the Glanville fritillary were used for electron microscopic analysis. Tissue samples were prepared by bisecting the thoraces and fixing the resulting slices in 2.5% glutaraldehyde (Fluka Chemie GmbH, Buchs, Switzerland), 1% formaldehyde (Electron Microscopy Sciences, Hatfield, PA, USA) in 100 mM Na-phosphate buffer (pH 7.4) for 2 h at room temperature, post-fixing in unbuffered non-reducing 1% OsO_4_ (Electron Microscopy Sciences, Hatfield, PA, USA) for 1 h on ice, and embedding in low viscosity resin (Agar Scientific Ltd., Essex, UK) according to standard protocols. Sections (60 nm thick) were cut using Leica Ultracut UCT ultramicrotome (Leica Mikrosysteme GmbH, Austria), and collected on pioloform coated single-slot copper grids, post-stained with uranyl acetate and lead citrate, and examined with Jeol EX1200 II TEM (Jeol Ltd., Tokyo, Japan) operating at 60 kV. Images were acquired with ES500W, model 782, CCD camera (Gatan Inc., Pleasanton, CA, USA) or with a film camera on the microscope.

For morphometric analyses, sections were cut from two blocks for each specimen, and three cells from each section were randomly chosen for imaging. Orientation of the block was adjusted to yield cross sections of the myofibrils, verified by the circular appearance of cross-sections of myofilaments. For each specimen, total areas of 145 µm^2^ and 67 µm^2^ comprising 18 images at the final magnifications of 19 000× and 28 000×, respectively, were analysed. To approximate differences in mitochondrial size and density, mitochondrial profiles of identical magnification were manually traced onto transparencies, which were scanned (Epson perfection 2400 Photo, model J111A, Seiko Epson Corp., Nagano, Japan). A dark or white line was defined to represent the outline of a profile. When a narrow space of around 40 nm in width was found between two areas of the same colour, the profiles were defined as separate. Cross-sectional area of mitochondria was measured as the percentage of the total area of the image using Image-Pro Plus (Media Cybernetics, Bethesda, MD, USA).

## Results

### Variation among Glanville fritillary populations in aerobic capacity

**Figure 2:**
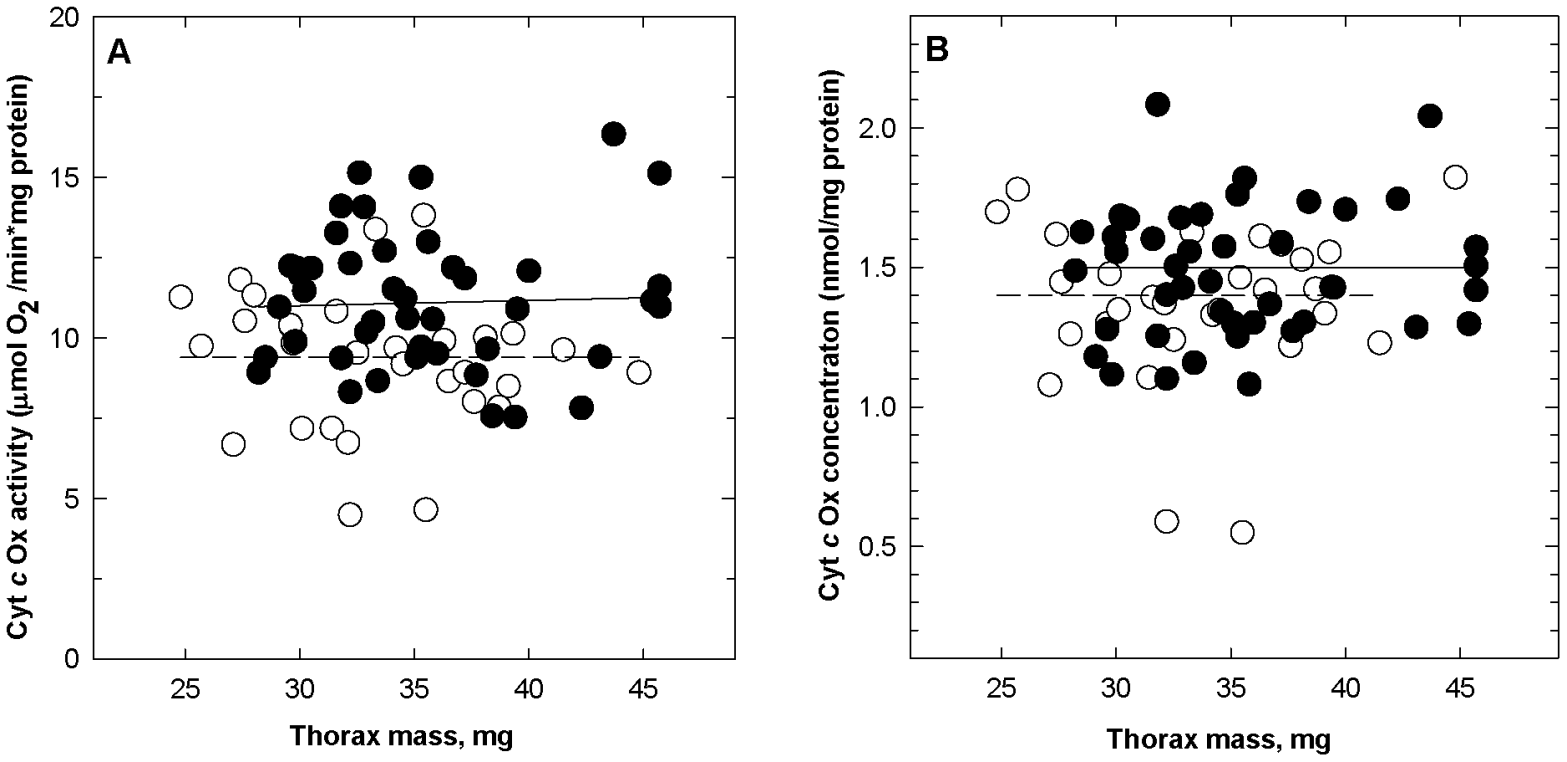
CytOx activity (A) and concentration (B) in flight muscles of Glanville fritillaries originating from old and new local populations. Results are shown for individuals from old (open symbols; *n* = 15 males, 13 females) and new populations (filled symbols; *n* = 19 males, 24 females), plotted against thorax wet mass. Mean CytOx activities and concentrations are shown as horizontal lines (solid lines, new populations; dashed lines, old populations). Statistics in text.

Mitochondrial CytOx activity was significantly higher in Glanville fritillaries originating from newly-established populations (11.2±0.4 µmol O_2_ per min per mg soluble protein; *n* = 43) than from old populations (9.1±0.4 µmol O_2_ per min per mg soluble protein, *n* = 28; Fig. 2A; one-way ANOVA, new *versus* old populations; *F_1,69_* = 5.9; *P* = 0.016). CytOx tissue content was slightly higher in individuals from new than old populations (1.5±0.02 versus 1.3±0.02 nmol CytOx per mg soluble protein, *n* = 44, 26; Fig. 2B; *F_1,69_* = 3.7; *P* = 0.05). In contrast, thorax soluble protein content did not differ between population types (55 versus 53 mg soluble protein per g thorax; *P*>0.05), nor were there differences in enzyme content or activity between males and females (*P*>0.4 for all comparisons). Finally, there was no difference in the CytOx activity or content between flight muscles of butterflies with different activity levels for the first 4–5 days of their adult life (*P*>0.1 for all comparisons).

Heterozygous individuals for SNP *Pgi_111* (AC) were more frequent in new than old populations (20 versus 3 individuals), whereas there was no difference in the numbers of the AA homozygotes (21 versus 25; Pearson *χ^2^* = 12.2, *P* = 0.002), as expected based on previous studies [4], [56]. CC homozygotes were rare, as has been observed previously for the Åland metapopulation [55]; there were only 2 individuals in this sample, both from new populations. CytOx enzyme activity and concentration did not differ among the common *Pgi* genotypes (P>0.5 for all comparisons).

### Variation among species in aerobic capacity

**Figure 3:**
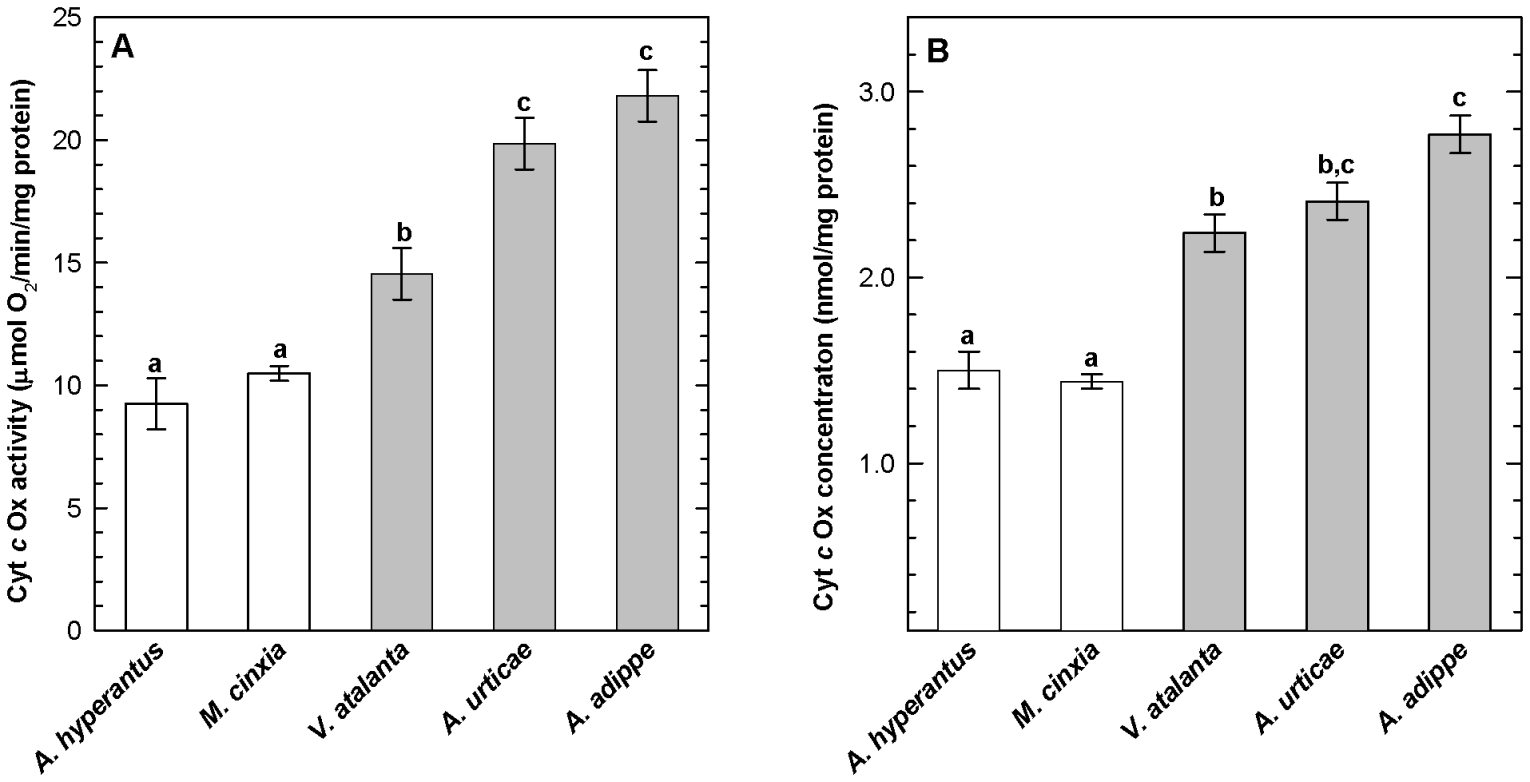
CytOx activity (A) and concentration (B) in flight muscles of five butterfly species with dissimilar flight behaviours. Means (±SE) of CytOx activity (A) and concentration (B) for five species of butterfly, two short-distance fliers (open bars; Ringlet *Aphantopus hyperantus n* = 5; Glanville fritillary *M. cinxia n* = 71), and three strong fliers (shaded bars; Small Tortoiseshell *Aglais urticae n* = 5; High Brown fritillary *Argynnis adippe n = 5*; Red Admiral *Vanessa atalanta*, *n* = 15). Letters denote differences between species (Tukey's HSD). Statistics in text.

Flight muscle CytOx enzyme activity and tissue concentration differed significantly among the five Nymphalid butterfly species. CytOx enzyme activity and concentration were lower in the short-distance flyers, the Glanville fritillary (*M. cinxia*) and Ringlet (*A. hyperantus*), than in the strong, long-distance flyers, the Tortoiseshell (*A. urticae*), High Brown fritillary (*A. adippe*) and Red Admiral (*V. atalanta*) (Fig. 3; nested ANOVA; species [flight type]: enzyme activity: *F*_3,95_ = 16.1, *P*<0.0001; concentration: *F*_3,95_ = 4.2, *P* = 0.008). Thorax soluble protein differed among species (Table 1; nested ANOVA; species [flight type]: *F*_3,95_ = 14.1, *P*<0.0001), but not between short- and long-distance fliers (*P*>0.5).

### Density of mitochondria in flight muscles

**Figure 4:**
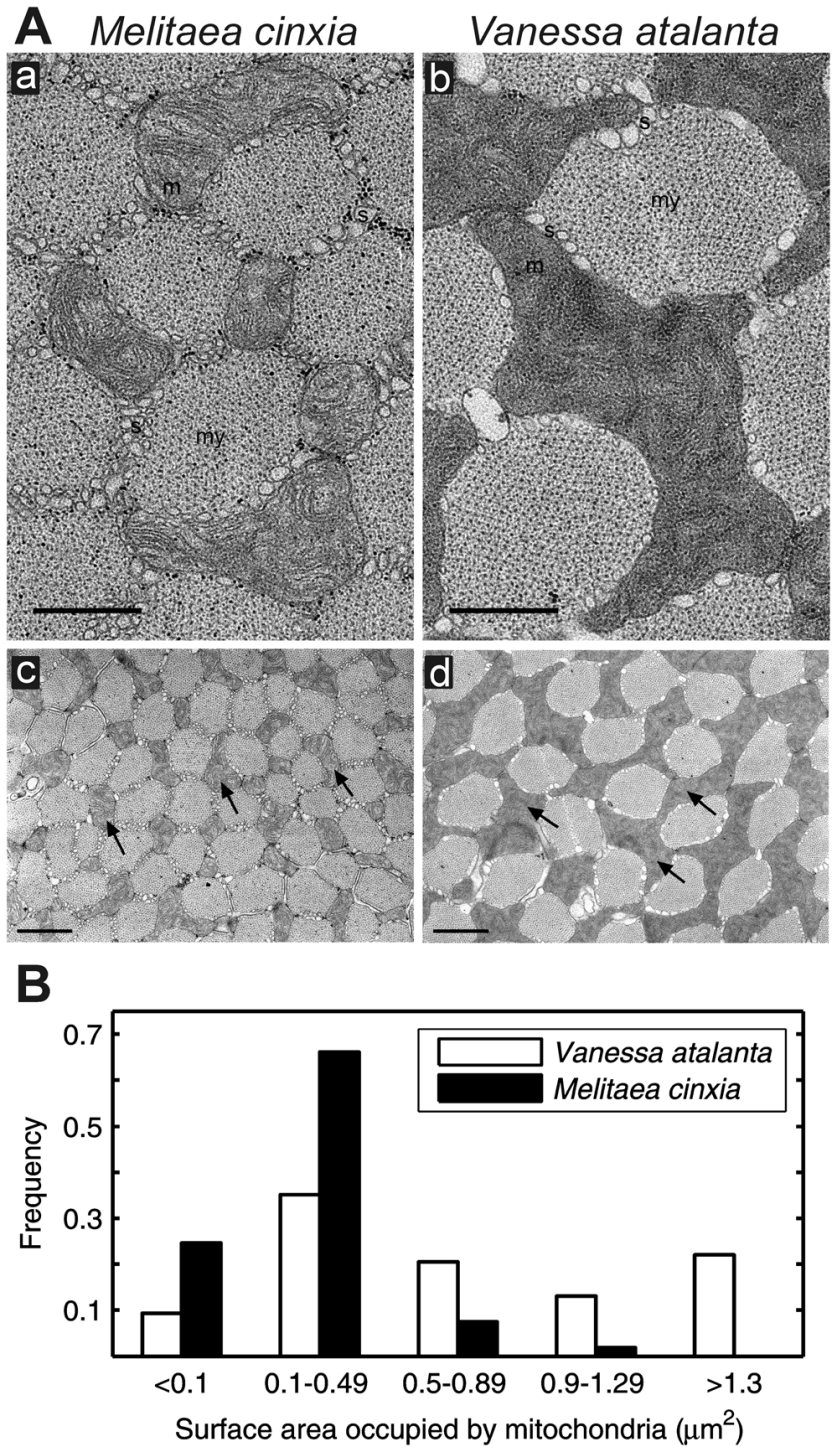
Mitochondrial structure and density in the flight muscles of two butterfly species with dissimilar flight behaviours. (A) Microscopic images of flight muscle of the Glanville fritillary (a, c) and the Red Admiral (b, d). Mitochondria (m and arrows), sarcoplasmic reticulum (s) and myofibrils (my) are indicated. Bars are 500 nm in a and b, 1 µm in c and d. (B) Distribution of mitochondrial profiles from thin sections of the flight muscle.

To complement the above spectroscopic results, we carried out a limited microscopic study of the flight muscles of the Glanville fritillary and the Red Admiral to discover whether there are structural differences in mitochondria consistent with differences in flight behaviour and biochemical indicators of muscle aerobic capacity (Fig. 4). There were no significant overall differences in cell size between the species. However, myofibrils, which are clearly defined by the surrounding sarcoplasmic reticulum in the flight muscle of both species, are smaller in the Glanville fritillary than in the Red Admiral. In addition, Glanville fritillary mitochondria appear to have less dense cristae than those of the Red Admiral (Fig. 4A a,b). Total sarcoplasmic volume occupied by mitochondria was higher in Red Admiral flight muscle (33%) than in that of the Glanville fritillary (24%) (Student's *t*-test; *P*<0.0001). Furthermore, mitochondria have a larger cross-sectional area (0.99 µm^2^ versus 0.24 µm^2^) and are more uniformly distributed throughout the myofibril matrix in Red Admiral than Glanville fritillary flight muscle (*P*<0.0001; Fig. 4A c,d). In the Red Admiral, flight muscle mitochondria are larger and more abundant than in the Glanville fritillary. A histogram of the surface area profile shows that 48% of thin section profiles in the Glanville fritillary fall between 0.1 to 0.29 µm^2^, and none had an area greater than 2 µm^2^, whereas in the Red Admiral profiles were present in all size categories, and 12% were greater than 2 µm^2^ in area.

## Discussion

Flight is one of the most energetically demanding activities performed by insects and is critical for dispersal; thus, it is expected that selection will act strongly on components of flight metabolism, including flight muscle aerobic capacity. Here we examined differences in aerobic capacity between populations and among species of butterflies with dissimilar dispersal abilities and flight behaviours. We used biochemical indicators of aerobic capacity known to vary in other species of flying animals: the content and activity of the mitochondrial enzyme Cytochrome-*c* Oxidase (CytOx), and the size and number of mitochondria in flight muscle. We show that aerobic capacity differs among local populations of the Glanville fritillary characterized by distinct dispersal abilities and flight metabolic rates. We also show that the flight muscle of the Glanville fritillary has relatively low aerobic capacity and mitochondrial content in comparison with species of butterflies known to be strong or long-distance fliers.

### Differences in aerobic capacity among Glanville fritillary populations

CytOx catalyses a four-electron reduction of molecular oxygen to water, a reaction in which inspired oxygen functions as a sink for electrons delivered through the complexes of the respiratory chain to CytOx. We found that CytOx activity was higher in Glanville fritillary butterflies from newly established than old local populations (Fig. 2A). Furthermore, though there is much variation in CytOx activity in both population types, most of the lowest CytOx activities were measured in individuals from old populations. These results are consistent with previously reported higher flight metabolic rates in individuals from newly established populations [5], [26], [27]. In those studies, flight metabolic rate was estimated by measuring carbon dioxide production, which is directly proportional to oxygen consumption during aerobic metabolism in insects [46]. Taken together, these results suggest that butterflies in new populations have higher aerobic capacities than those from old populations.

Previous studies of the Glanville fritillary in the Åland Islands metapopulation have demonstrated that heterozygous individuals at the SNP *Pgi_111* are more effective dispersers than *Pgi_111* homozygotes, thereby highlighting the role that glycolysis may play in dispersal ability [4], [5], [23], [26], [27], [56]. Spatial variation in *Pgi* genotypes observed in this study was consistent with previous results, with higher frequency of the *Pgi_111* heterozygotes in newly established populations, but we did not observe a correlation between CytOx activity and *Pgi* genotype. The relatively small number of *Pgi_111* heterozygotes found in old populations makes multi-gene comparisons challenging. In another insect species, the Sierra willow beetle *Chrysomela aeneicollis*, functionally significant variation in CytOx activity has been observed among *Pgi* genotypes [51]. Recent genomic studies of the Glanville fritillary suggest the possibility of epistasis between *Pgi* and other metabolic genes [47], including those coding for mitochondrial metabolic enzymes [50]. Expression patterns of a number of these genes differ between old and new populations, including *Sdhd*, which encodes for the mitochondrial enzyme succinate dehydrogenase. The frequency of the *Sdhd* D allele is significantly higher in new than old local populations, and butterflies carrying *Sdhd* D have higher gene expression of carbohydrate metabolism genes [47]. Thus, there are a number of mechanisms by which *Pgi* may directly or indirectly influence the activity of other metabolic proteins, resulting in the strong natural selection observed in the Glanville fritillary and other insect species [57].

### Differences between butterfly species in aerobic capacity and mitochondrial density

We found large differences in both CytOx activity and content between five species of butterflies examined in this study (Table 1; Fig. 3). The Glanville fritillary (*M. cinxia*) and the Ringlet (*A. hyperantus*) had much lower aerobic capacities than the three species known to be strong or long-distance flyers, the Tortoiseshell (*A. urticae*), High Brown fritillary (*A. adippe*) and Red Admiral (*V. atalanta*). One explanation for differences between the species is that strong, long-distance fliers have higher mitochondrial concentrations and higher packing density of cristae than short-distance fliers, rather than a higher density of mitochondrial enzymes *per se*, as observed in flight muscle of other insects [16], [58]. Glanville fritillary mitochondria appear to be smaller and occupy less muscle cell volume than Red Admiral mitochondria (Fig. 4), consistent with this hypothesis. The powerful fliers studied here have over 50% more CytOx in their flight muscles than the Glanville fritillary or Ringlet, suggesting that butterflies flying long distances may have a higher capacity for aerobic energy metabolism in their flight muscles than short-distance fliers. Differences in carbohydrate and fatty acid metabolism may also play a role in the observed differences in mitochondrial activity and content between species. In many insects, carbohydrates are oxidized in short duration flights, but fatty acids fuel flights of longer duration, and much of this fatty acid catabolism occurs in the mitochondria [43]. Thus, higher mitochondrial concentrations observed in the strong fliers may be associated with higher capacities for fatty acid utilization during long distance flight. Unfortunately, activities of enzymes directly indexing fatty acid metabolism were not measured in the present study.

The five species examined here fall into two contrasting groups in terms of flight behaviour, two species being weak fliers, the Glanville fritillary and Ringlet, while the other three species are all powerful, fast fliers, with the Red Admiral being even a transcontinental migrant. They differ in other life history characteristics as well. The Red Admiral has two generations per year, the others all have one generation, and the Small Tortoiseshell overwinters as an adult. The two weak flyers are smaller than the other species, which is a common though not a universal correlate of general mobility in butterflies. While we cannot exclude the possibility that these other life history differences among the species influence flight muscle CytOx content and enzymatic activity, the most parsimonious explanation of our results is that the metabolic differences are associated with the differences in mobility and flight capacity.

## Conclusion

The present results imply that the modest aerobic capacity of the Glanville fritillary may restrict the range of its dispersal. At the same time, individuals with even slightly elevated aerobic capacity, as found here in butterflies from newly-established populations, may have an advantage in dispersing to and colonizing new areas. Selection on aerobic capacity in this and many other butterfly species may become increasingly significant with climate change and habitat fragmentation, which make population persistence in fragmented landscapes more tenuous.
